# Change in self-reported emotional distress and parenting among parents referred to inpatient child psychiatric family treatment

**DOI:** 10.3109/08039488.2011.630752

**Published:** 2011-11-16

**Authors:** Tormod Rimehaug, Turid Suzanne Berg-Nielsen, Jan Wallander

**Affiliations:** Assistant professor, Regional Centre for Child and Adolescent Mental Health, Medical School, Norwegian University of Science and Technology (NTNU) and Child and Adolescent Psychiatry Department, Nord-Trondelag Health Trust; Associate professor, Regional Centre for Child and Adolescent Mental Health, Medical School, Norwegian University of Science and Technology (NTNU); Professor, Regional Centre for Child and Adolescent Mental Health, Medical School, Norwegian University of Science and Technology (NTNU) University of California, Merced, California, USA

**Keywords:** Authoritarianism, Internalized problems, Protectiveness, Treatment-as-usual, Warmth

## Abstract

Rimehaug T, Berg-Nielsen TS, Wallander J. Change in self-reported emotional distress and parenting among parents referred to inpatient child psychiatric family treatment. Nord J Psychiatry 2011;64:1–8.

*Aims*: Our aim was to examine changes in distress symptoms and parenting dimensions among parents in child psychiatry services (*clinic parents*) (*n*= 102). Parents were followed from referral and admission to 3-month and 12-month follow-ups of “treatment-as-usual” at inpatient *family clinics*. These measurements were compared with a sample of *community parent* (*n* = 439) standards. *Methods*: Standardized questionnaires measuring the child's problems, parental anxiety and depression symptoms (distress), and warmth protectiveness and authoritarianism (parenting dimensions), were distributed to parents four times (T0–T1–T2–T3). The family clinics received families whose children had long-term problems and unsatisfactory previous treatment outcomes. *Results*: Clinic mothers, but not fathers, showed an improvement in distress symptoms at the 3-month (T2) and 12-month (T3) follow-ups relative to at admission (T1). Nevertheless, clinic mothers displayed distress symptoms at all measurement points compared with community parents. Parents of children with learning/developmental problems and attention disorders showed significantly higher warmth scores at the 3-month and 12-month follow-up compared with at admission, although the levels remained lower than those of community parents. In contrast, parents of children with emotional problems showed the same level of *warmth* as community parents and lower levels of *protectiveness*, but no change in these parenting dimensions T1–T2. *Implications*: Parental emotional distress symptoms and parenting characteristics should be addressed systematically in child psychiatry to inform evaluations of the context of the child's problems and the family's treatment needs. Systematic and effective treatment components related to parenting should be implemented.

The heterogeneous population of parents with children receiving psychiatric treatment, denoted here “clinic parents”, is rarely the primary target of research. Yet, it is commonly accepted that caring for children with prolonged psychiatric problems can be emotionally taxing and burdensome. As many as one out of ten parents experience burdens in their parenting role because of their child's mental health problems (1), and many parents of these children are known to suffer from self-blaming (2) and emotional distress (3) increasing with the severity and duration of problems and when externalizing problems are present (4, 5). Whereas the influence of children's chronic and serious *somatic* illness and intellectual disability on their parents has been studied well (6, 7), relatively little has been documented for parents with children who have psychiatric problems.

Traditionally, parents' emotional distress and parenting problems have primarily been viewed as risk factors when observed together with a child's problems (1), although clinicians are increasingly aware of the bi-directionality of effects between child and parent (8). Utilizing parents as resources in treatment and aiding them in improving their parenting requires knowledge about clinic parents as a heterogeneous group. The present knowledge about these parents is insufficient. The aim of the current study is to increase this knowledge.

Examining parents at selected time-points related to child treatment may increase our understanding of the interplay between child and parent functioning in families with children who have psychiatric problems. We examined parents before a waiting period, at admission to treatment, and at 3-month and 12-month follow-ups. Waiting for, entering and participating in treatment may alter distress levels and parenting. Changes at 3-month follow-up may be sustained or may deteriorate by the 12-month follow-up. However, in the naturalistic design of this study, the influence of situational, child and parental factors cannot be definitely separated.

Parents who accompanied their children in inpatient child psychiatric *family clinics* were chosen for this study because referrals to these clinics typically follow a long history of severe childhood problems with prolonged and unsuccessful treatment at previous service levels. Conceivably, these parents have experienced prolonged strain and distress (4, 5), and their functioning likely differs from that of community parents (8). Furthermore, the therapeutic programme is characterized by extensive parental involvement (9), increasing the probability of parental change.

Parenting dimensions, rather than specific behaviours, were chosen as a parsimonious representation of parenting (10) in this study. The dimensions of warmth, protective-ness and authoritarianism proposed by Kendler (11) based on dimensions previously developed by Parker et al. (12) have been shown to be relevant for child mental health (13, 14) and have been frequently used in research on psychiatric risks (8). Furthermore, these dimensions were suitable because of the availability of corresponding data on community parents in the area (15), and they have been found to differ between clinic and community parent samples (in part depending on gender) in our previous research (unpublished data). These results also strengthened the motivation for including gender differences and interactions in the analytic strategies (16).

Anxiety and depression symptoms were chosen because they indicate distress as well as possible clinical conditions (17). Associations between parenting, parent psycho-pathology and child problems shown in previous studies (13) motivated the exploration of whether the results were confined to parents of children with specific diagnoses.

## Aims

The aim of this study was to examine changes in self-reported parenting dimensions and levels of emotional distress among parents of children who were referred to child psychiatric family inpatient clinics. Changes between referral, admission and 3-month and 12-month follow-ups were examined, and levels contrasted to community parent standards. We also examined whether changes in parenting dimensions and emotional distress were related to one another, parental gender, demographics, or child problems and diagnosis. Although changes across time were assessed, this naturalistic study did not aim to evaluate treatment effect.

## Materials and Methods

### The clinic parent samples

**Figure 1:**
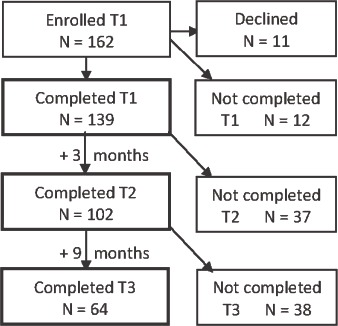
Participant flowchart T1–T2–T3.

The clinic parents were recruited from families referred to three child psychiatric family inpatient units (denoted *family clinics* in the following). Referred parents were consecutively invited to participate during a 4-year period. The catchment area included two cities, several small towns and large rural areas. Of 160 eligible parents, 151 gave their informed consent to participate. Of these participants, 139 completed the main data collection at admission (T1) to the family clinic (87% of those eligible), and 102 completed the 3-month follow-up after discharge at T2 (representing 64% of those eligible). Of these 102 participants, 64 (40% of the originally eligible, 61% of T2 participants) completed the 12-month follow-up T3 assessment. Because of practical problems, as often occurs in naturalistic settings, the T0 measurement was only collected from a sub-sample of 15 out of the *last* 16 parents invited into the study (representing 9% of those eligible, 15% of T2 participants). Thus, the T0 measurement could only be used as a supplementary exploration of the stability of the T0–T1 responses and is not included in the flowchart Fig. 1. T0 took place in connection to referral, prior to a waiting period that averaged 8 weeks.

Written parental consent forms were obtained by clinicians after distribution of written information and invitation. Questionnaires were distributed by clinicians and returned to research assistants at the family clinics in sealed envelopes.

Demographic information is shown in Table 1. The referred children had already received community services for an average (± standard deviation) of 3.9 ± 2.2 years (range 0.5–10 years) prior to receiving services in outpatient child psychiatric clinics for an average of 1.8±1.8 years (range 0.5–10 years). Their total service history was thus, on average, 5.8±2.9 years (range 1–12 years) before referral to the family clinic. On the Child Behaviour Checklist, the sample mean scores at referral for each problem subscale were in the moderate clinical range (T-score mean = 68.7±8.1) for all subscales.

**Table 1:** Demographics of clinic and community parents and their children.

	Clinic parents (T1–T2) *n* = 102	Community parents, *n* = 439
Parental gender		
Mothers	65% (*n* = 66)	59% (*n* = 257)
Fathers	35% (*n* = 36)	41% (*n* = 182)
Parental age		
Mean (*s*)	39.6 (7.4)	40.6 (5.6)
Mothers	38.7 (7.1)	39.5 (5.2)
Fathers	41.6 (7.8)	42.0 (5.7)
Child's gender*		
Boys	67%	55%
Girls	33%	45%
Child's age		
Mean (*s*)	10.8 (2.7)	11.4 (2.9)
Range (years)	7–15	8–15
Offspring number, Mean (*s*)	2.8 (1.1)	2.6 (0.9)

*s*, standard deviation.

* Only the child gender balance was significantly different between the samples.

### Clinical service and diagnostic evaluation

The family clinics admit families for a 2–4-week stay. Each family member participates in a concentrated full day schedule, including assessment of the child and treatment for the child and family (9, 18). Standardized child assessment instruments are used, while the treatment programmes are non-standardized, non-manualized and eclectic in their nature, which is representative of “treatment-as usual” in child psychiatry in Norway. Parents' emotional distress or other psychiatric symptoms were not specifically targeted in the assessment or treatment. Families offered this service represent approximately 1% of the cases referred to child psychiatric treatments facilities in the area. Clinicians tend to refer families to this service when family members have lasting and/or multiple difficulties within somatic, addiction, marital, social and economic areas in addition to psychiatric problems.

Because of differences in clinical routines not influenced by this study, only two of the three family clinics obtained diagnoses for children according to clinical assessments based on ICD-10 criteria: grouped in the following five broad, non-exclusive categories: 1) emotional disorders (F30–42, F50, F92–94), *n* = 32; 2) attention disorders (F90), *n* = 31; 3) learning and developmental disabilities (F70–89), *n* = 27; 4) conduct disorders (F91–92), *n* =12; 5) trauma-related disorders (F43–44), *n* = 8. In total, 26% of the children were assigned to two of these categories (comorbidity).

### Community parent sample

The community parent sample was included to represent standards regarding parenting dimensions and distress symptoms. Community parents were invited to participate from 20 public schools, of which 12 schools enlisted 606 eligible parents for the study. Teachers distributed the questionnaires to children in sealed envelopes to be taken home and completed by their parents. The questionnaires were then returned by mail to the principal investigator, and 439 parents responded, 73% of those enlisted. The recruitment area overlapped largely with the catchment area for the inpatient family clinics, a summary of the community parent demographics is shown in Table 1, and in previous research (15).

## Methods

### Instruments

*Parenting dimensions* were measured with the Parental Bonding Instrument (PBI) as revised by Kendler (11) based on an original version from Parker et al. (12), including the dimensions of Warmth (seven items) about positive emotions and empathic communication, Protectiveness (five items) tapping infantilization and monitoring and Authoritarianism (four items) concerning constraints on the child's freedom and autonomous choices. Kendler's revision of the original PBI (12) also included a version for self-reported *current parenting* practices toward offspring, which was used in this study based on approved item translations (12, 19).

*Anxiety and depression symptoms* were measured with the Hospital Anxiety and Depression Scales (HADS) (17, 20). HADS produces separate scores for anxiety (seven items) and depression (seven items). A score of 8 has been used in several previous studies as a minimum cutoff for clinical levels of symptoms (20), and the rate of this is used here only for descriptive purposes. Detailed accounts of psychometric properties can be found elsewhere (21). The concepts of anxiety and depression as used in this paper refer to symptoms and symptom levels and not to diagnostic categories.

*Child problems* as described by parents were measured by the Child Behaviour Checklist (CBCL) problem scales, Norwegian version (22), consisting of 120 items forming eight subscales and the subtotals of the Internalizing Problems scale and Externalizing Problems scale. The CBCL is part of a broader multi-informant assessment battery of competencies and emotional/behavioural problems, the Achenbach System of Empirically Based Assessment (ASEBA) (23).

### Statistics

All analyses were done in SPSS 17.0 unless otherwise specified. All variables were converted to *z*-scores relative to gender, based on the community sample (mean = 0.0±1.0) to allow direct comparison of differences and levels in a common metric. A *z*-score of —1.0 based on the Warmth subscale was used as the criterion for “low warmth” for illustrative purposes.

Change over time in the clinic sample for continuous variables was tested using GLM (General Linear Models) repeated measurements. Because of a small T0 sample and T3 drop-out, T0–T1 and T2–T3 longitudinal differences were analysed separately from T1–T2. Measurements were not repeated with community parents. Gender differences and differences between clinic parents and community parents for continuous variables are reported from two-way gender X sample analysis of variance (ANOVA) GLM models.

Associations between continuous variables were analysed with Pearson's product-moment correlations in SPSS 17.0. Owing to the high number of tests for demographic variable correlations to parenting and to the levels and changes in parental emotional problems (>100), the threshold for statistical significance of these values was set at *P*< 0.01.

Because of the existence of non-exclusive categories (comorbidity), parents of children in different diagnostic groups could not be directly compared but parents in each of these diagnostic categories could be compared with community parents. The low number of subjects in two diagnostic groups (conduct disorders and trauma-related disorders) and T2–T3 drop-out limited the statistical power for some comparisons, especially those related to T3.

Multilevel analyses were not required in this study because emotional problems and parenting were not significantly different between the sampling clusters for clinic parents (clinics) or for community parents (schools) and were not correlated between mothers and fathers within families.

## Results

With the exception of parent gender proportions, there were no significant differences between the clinic and community parents with regard to demographic information (Table 1). There were no significant T1 differences in demographic, parenting or emotional variables between participants and drop-outs at T2 or T3, or between T0 participants and those *not* invited for T0 measurements.

### Parental emotional problems before and after treatment

**Figure 2:**
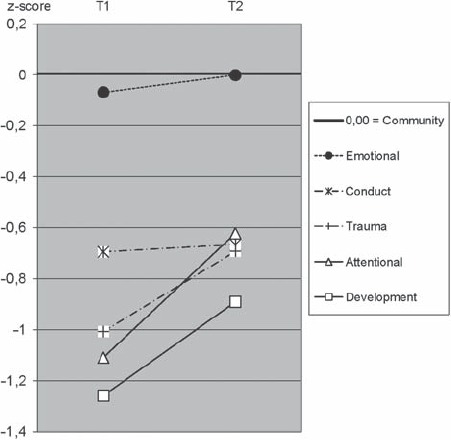
Parental differences between T1 admission and T2 3-month follow-up using PBI Parenting Warmth scores within non-exclusive child diagnostic groups. Solid lines indicate: 1) a significant T1–T2 difference and 2) a significant deviations in level from the community sample. Broken lines indicate: 1) a non-significant T1–T2 difference and 2) a significant level of deviation from community parents. Dotted lines indicate: 1) a non-significant T1 – T2 difference and 2) non-significant deviations in level from the community sample.

Anxiety and depressive symptoms among clinic mothers but not fathers were significantly reduced from T1 to T2 and remained lower at T3. For both anxiety and depression, *clinic mothers* but not fathers, scored higher than community parents at all four points T0–T1–T2–T3 from referral to 12-month follow-up. These differences between clinic and community parents did not depend on child diagnostic groups. Details of these statistical analyses are presented in Table 2. Levels and changes between T1–T2 are illustrated in Fig. 2.

**Table 2:** Differences in anxiety, depression and parenting warmth in clinic parents over time, and contrasted to community parent standards.

Mothers only	T0	T1	T2	T3
Anxiety				
Contrast clinic/community	Mean = 1.30z, *F*(1,261) = 9.42**	Mean = 1.12z, *F*(1,318) = 55.33**	Mean = 0.66z, *F*(1,316) = 17.87**	Mean = 0.57z, *F*(1,293) = 10.16**
Clinic change relative to T1	*ns*	−	Δ*M*_1−2_ = −0.46z; *F*_1−2_(1,58) = 16.34**	Δ*M*_1−2_ = −0.72z; *F*_1−3_(1,36) = 23.97**
Depression				
Contrast clinic/community	Mean = 1.22z, *F*(1,262) = 10.13**	Mean = 1.07z, *F*(1,318) = 49.58**	Mean = 0.51z, *F*(1,316) = 11.02**	Mean = 0.68z, *F*(1,293) = 12.56**
Clinic change relative to T1	*ns*	−	Δ*M*_1−2_ = −0.59z; *F*(1,58) = 16.34**	Δ*M*_1−2_ = −0.74z; *F*(1,36) = 15.45**

All parents	T0	T1	T2	T3
Warmth				
Contrast clinic/community	Mean = −1.30z, *F*(1,448) = 13.54**	Mean = −0.90z, *F*(1,522) = 18.04**	Mean = −0.46z, *F*(1,522) = 9.93**	Mean = −0.42z, *F*(1,489) = 8.21**

Only significant differences or changes have been included, based on analysis of variance. No signifi cant differences or changes among fathers.

**P < 0.005, * P < 0.05, T0 = Referral, T1 = admission, T2 = 3-month follow-up, T3 = 12-month follow-up, Δ*M*_1−2_ = Mean change T1 − T2.

Illustrating these mean score changes by corresponding prevalence rate changes, showed that clinical level anxiety symptoms among clinic mothers decreased from 66% at T1 to 50% at T2 and then 45% at T3, compared with the 19% standard rate in the community sample. The prevalence of clinical-level depression symptoms among clinic mothers decreased from 44% at T1 to 16% at T2 and 24% at T3, as compared with 6% in the community sample.

### Parenting dimensions before and after treatment

Change from T1 to T2 and level *at* T1 and T2 of Warmth differed among clinic parents relative to child clinical diagnoses. Because of the small number of participants in several diagnostic subgroups, T0 and T3 were not analysed when separating parents by child diagnostic category.

Parents of children with learning and developmental disabilities and parents of children with attention disorders showed significantly increased Warmth from T1 to T2. Parents of children with conduct disorders and posttrau-matic problems did not exhibit significant change in parenting from T1 to T2; however, these groups were small (*n* = 12 and *n* = 8, respectively), and thus the power to detect or reject changes in Warmth was not sufficient.

Parents of children with learning and developmental disabilities and parents of children with attention disorders showed lower Warmth levels than community parent standards at both T1 and T2 despite the improvements. Parents of emotionally troubled children did not change their Warmth level significantly across T1–T2, and did not differ from community parents at either T1 or T2.

**Figure 3:**
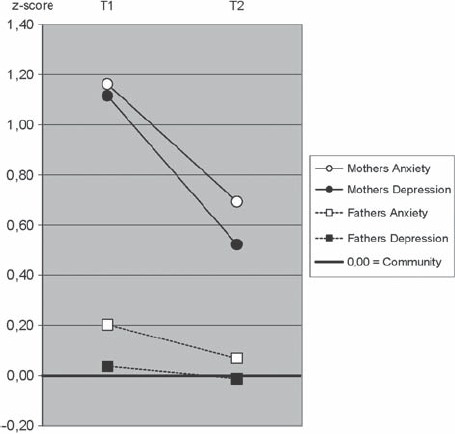
Parental differences between T1 admission and T2 3-month follow-up using HADS Anxiety and Depression scores. Solid lines indicate: 1) significant T1–T2 difference and 2) significant deviations in level from the community sample. Dotted lines also indicate: 1) a non-significant T1–T2 difference and 2) non-significant deviations in level from the community sample.

Protectiveness did not change significantly across T1–T2, but showed significantly lower scores compared with community parents only among parents of children with emotional problems at both T1 and T2. Authoritarianism did not change significantly across T1–T2, but showed significantly higher scores only among parents of children with learning problems at T2 and among parents of children with attention disorders at both T1 and T2. Details of these analyses are shown in Table 3, and the main results are illustrated in Fig. 3.

**Table 3:** Differences in parenting dimensions in clinic parents over time and contrasted to community parent standards, according to clinical child diagnoses.

	PBI Parenting Warmth			
	T1 and T2 clinic parents		Change in clinic parents	
Child clinical diagnoses	Contrasted to community parents	T0 – T1	T1 – T2	T2 – T3
Emotional disorders	*M*_1_ = −0.07 ns; *M*_2_ = 0.00 ns	ns	ns	ns
Attention disorders	*M*_1_ = −1.11, *F*(1,458) = 28.55**; *M*_2_ = −0.62, *F*(1,458) = 9.57**	ns	Δ*M*_1–2_ = 0.40, *F*(1,22) = 16.38**	ns
Learning/develop-mental disorders	*M*_1_ = −1.26, *F*(1,454) = 27.80**; *M*_2_ = −0.89, *F*(1,454) = 14.32**	ns	Δ*M*_1–2_ = 0.37, *F*(1,63) = 5.69**	ns
Conduct disorders	*M*_1_ = −0.69, *F*(1,446) = 5.08*; *M*_2_ = −0.67, *F*(1,446) = 4.73*	ns	ns	ns
Post-traumatic problems	*M*_1_ = −1.01, *F*(1,443) = 7.68*; *M*_2_ = −0.69, *F*(1,443) = 3.72*	ns	ns	ns

PBI Protectiveness				
Emotional disorders	*M*_1_ = −0.38, *F*(1,463) = 3.86*; *M*_2_ = −0.42, *F*(1,463) = 4.68*	ns	ns	ns
All other disorders	*M*_1_ = −0.09 ns; *M*_2_ = −0.20 ns	ns	ns	ns

PBI Authoritarianism				
Attention disorders	*M*_1_ = −0.94, *F*(1,460) = 19.23**; *M*_2_ = −0.62, *F*(1,460) = 8.44**	ns	ns	ns
Learning/develop-mental disorders	T1 ns; *M*_2_ = 0.51, *F*(1,455) = 4.59*	ns	ns	ns
Other disorders	*M*_1_ = 0.15 ns; *M*_2_ = 0.24 ns	ns	ns	ns

Only significant differences or changes have been included, based on −3. **P <0.005, 8*P* < 0.05. T1 = admission, T2 = 3-month follow-up, *M*_1_ = Mean T1, *M*_2_ = Mean T2, Δ*M*_1−2_ = Mean change T1–T2.

### Correlations of changes in clinic parents' emotional distress and parenting

The levels and changes of parenting dimensions or of emotional distress symptoms during any period were not statistically associated with gender or demographic information for parents or children.

The analyses of associations between change in emotional distress and change and level of parenting dimension showed that reduction in parental depression across T1–T2 correlated with increased Warmth from T1 to T2 (*r*_1–2_ = 0.32, *P*<0.01), but not in the other two parenting dimensions. Parental anxiety reduction across T1–T2 correlated with lowered Protectiveness (*r*_1–2_ = 0.29, *P* < 0.01). In agreement with this result, depression scores correlated negatively with Warmth at both T1 and T2 measurement points (*r*_1_ = – 0.23 and *r*_2_ =–0.30, *P*<0.01), and anxiety correlated positively with Protectiveness at T2 (*r*_2_ = 0.33, *P*<0.01).

Reduction in maternal Anxiety, but not Depression, from T1 to T2 showed positive correlations with improved scores on both the CBCL Child Externalizing and Internalizing Problems subtotal scales T1–T2 (*r*_i_ = 0.33, *P*< 0.05 and *r*_x_ = 0.44, *P* < 0.01).

## Discussion

The aim of the present study was to examine changes in parenting and symptoms of parental emotional distress across periods related to treatment in child psychiatric family clinics. There was a significant reduction in maternal distress symptoms after child “treatment-as-usual”. This lower level of distress was maintained during the 3–12-month follow-up interval, but was still significantly higher than community parent standards. Parental warmth improved significantly among parents of children with learning and developmental disabilities and attention disorders after treatment, but was still significantly lower than among community parents at follow-up. In contrast, parents of children with emotional disorders showed no change, and had similar warmth and lower levels of protectiveness compared with community parents. Increase in parental warmth and improved depression was associated, and so was reduced parental protectiveness and improved anxiety. The reduction in parental anxiety was correlated with reductions in parental accounts of child problems across the treatment period, but the reduction in parental depressive symptoms were not.

### Parental emotional distress

The subsequent improvement in distress symptom after treatment suggests that child problems or parental perceptions of child problems may have an important influence on the parents' symptoms, because the problems as well as the perceptions of them may have changed during treatment, but likely changed little during the waiting period. The association between the reduction in maternal anxiety and lower child problem scores supports this interpretation. However, lower parental anxiety may also result in a more positive evaluation of child problems.

The significant change in maternal anxiety and depressive symptoms across the treatment period can be interpreted in several ways. The admission of the family into the inpatient clinic could have represented a situational stress that increased symptoms at T1, or a relief that the waiting period had finally ended. However, the consistent level of distress symptoms between the time of referral and admission (T0–T1) suggests that such situational factors cannot explain the distress symptom levels at admission (T1).

The consistently elevated symptom levels compared with community parents at all measurement points suggest that parental problems and disorders at clinical or subclinical levels may explain some of their distress symptom levels. The pre-treatment symptom rates in the present sample were 1.5 and 2.4 times higher than those reported for mothers of paediatric diabetes patients (also using HADS) (24). Even at the follow-ups, the rates of anxiety and depressive symptoms in the current study were two and four times that of community parents. The increased maternal psychiatric vulnerability and morbidity indicated by these results could represent a pathogenic factor for the child, but may also reflect genetic or situational risk factors shared by parent and child (25).

### Parenting within child diagnostic groups before and after child treatment

The results suggest that the child's diagnostic category was an important moderator of the change and level of parenting dimensions. The unchanging and “normal” levels of parental warmth and the lower score on protective-ness among parents of emotionally disturbed children are in contrast to previous studies that reported *lower* warmth and *higher* protectiveness and/or authoritarianism among these parents (14). A possible explanation for this atypical result is that the families in the current study had a prolonged clinical history that included community and outpatient interventions. Parents of children with internalized problems may have gained a greater benefit more from these interventions than those of children with other problems. In addition, internalized problems are known to represent less parental burden (4), although any child disorder may challenge parental care and require increased levels of protection and/or authority (8).

A significant increase in parental warmth after treatment was shown among parents of children with developmental and attention disorders (disorders for which parental warmth is an unlikely pathogenic factor). The low parental warmth in most diagnostic groups before treatment may have resulted partly from the long-term burden of care and worries about the troubled child. The subsequent increase in warmth after treatment may have arose from improved parental hope and understanding, and diminished concerns and daily strains, although no association between child improvement and increased warmth was found.

Low warmth may still have preceded and contributed to functional impairment and the development of secondary problems even among children with these disorders (10, 26); however, the finding of increased warmth after treatment does not correspond with this assumption. Nevertheless, even the parent groups that showed improvement still displayed significantly less warmth than community parents at the follow-ups, suggesting that low parental warmth is not merely a result of child problems. Parental characteristics that might contribute to lower warmth in the parent–child relationship include personality traits, mental health problems and attachment insecurity (8, 25), all of which are known to increase the general risk of child maladjustment (14).

According to previous research, low parental warmth is most likely to have contributed to the development of child conduct disorders (10). The child conduct disorder group demonstrated low warmth before treatment and no indication of improvement after treatment. This is consistent with previous research that shows that child psychiatric “treatment-as-usual” is unlikely to significantly change parent or child behaviour in families with conduct disorders (10).

### Strengths and limitations

Comparison of the clinic samples of parents with a representative community sample, and the repeated evaluations of clinic samples recruited at three different family clinics, are strengths of this study. The clinic parent sample, however, was highly selected for children who had received many years of service prior to enrolment in the family clinics. Thus, these parents are not representative of child psychiatric clinic parents in general, but they do represent unsuccessfully treated cases. Such cases are routinely encountered in clinics but are very rarely the focus of research.

The group mean results may conceal subgroup variation and individual patterns that the present design and analytic strategy could not detect.

The use of clinical diagnoses limits the reliability of classification. The small size of some diagnostic groups is a consequence of the naturalistic design and impedes some statistical comparisons. Furthermore, the relatively low prevalence of comorbidity can be questioned in this sample, where considerable severity and chronicity of the children's problems should be expected. The small T0 sample and the T3 drop-outs threatened the power of our statistics to evaluate these two measurement points properly; however, there were no signs of systematic selection at T0 and T3, and the T0–T1 and T2–T3 differences were not only statistically non-significant but also quite small, supporting a no-change interpretation despite small samples.

### Implications

Interpretations and implications must be drawn with caution from a naturalistic study. However, from a community health perspective, the lower parental warmth and heightened levels of maternal distress may reflect a major burden for the parents of children with psychiatric problem and a prognostic risk for their children. Parental emotional distress may affect the validity of child assessment (27) and treatment prognosis (28) and may contribute to parenting problems (29, 30). Thus, parental distress and reduced warmth could prolong a child's problems in spite of effective individual treatment.

Our results motivate routine evaluation and monitoring of parental emotional distress and parenting dimensions when assessing and treating child psychiatric problems. This study did not intend to test treatment effects or infer causality, and parents who showed a positive change related to the treatment period could have been influenced by circumstances or events outside of the clinical setting and the treatment programme. Notably, the improvement in maternal distress and parental warmth appeared during a child- and family-oriented treatment that did not systematically address parental distress and parenting as part of the “treatment-as-usual” clinical services. Yet, family communication and mutual understanding were treatment components. Improvements were observed in this study despite exceptionally long histories of severe problems for the child.

Further research should investigate whether implementation of specific and systematic treatment components that focus on parents' emotional distress and parenting problems specifically related to child diagnoses may contribute to beneficial outcomes for parents as well as children.
